# Molecular conformation changes along the malignancy revealed by optical nanosensors

**DOI:** 10.1111/jcmm.12006

**Published:** 2013-01-10

**Authors:** Simona Cinta Pinzaru, Alexandra Falamas, Cristina Adriana Dehelean

**Affiliations:** a Faculty of Physics, Babes-Bolyai UniversityCluj-Napoca, Romania; b Faculty of Pharmacy, “Victor Babeş” University of Medicine and PharmacyTimişoara, Romania

**Keywords:** melanoma, SERS, skin tissues, plasmonic nanosensors, amino-functionalized nanoparticles

## Abstract

An interdisciplinary approach employing functionalized nanoparticles and ultrasensitive spectroscopic techniques is reported here to track the molecular changes in early stage of malignancy. Melanoma tissue tracking at molecular level using both labelled and unlabelled silver and gold nanoparticles has been achieved using surface enhanced Raman scattering (SERS) technique. We used skin tissue from *ex vivo* mice with induced melanoma. Raman and SERS molecular characterization of melanoma tissue is proposed here for the first time. Optical nanosensors based on Ag and Au nanoparticles with chemisorbed cresyl violet molecular species as labels revealed sensitive capability to tissues tagging and local molecular characterization. Sensitive information originating from surrounding native biological molecules is provided by the tissue SERS spectra obtained either with visible or NIR laser line. Labelled nanoparticles introduced systematic differences in tissue response compared with unlabelled ones, suggesting that the label functional groups tag specific tissue components revealed by proteins or nucleic acids bands. Vibrational data collected from tissue are presented in conjunction with the immunohistochemical analysis. The results obtained here open perspectives in applied plasmonic nanoparticles and SERS for the early cancer diagnostic based on the appropriate spectral databank.

## Introduction

Raman spectroscopy has currently become a powerful vibrational technique largely used to probe the molecular composition of biological tissues [1–4]. Raman spectra provide information on molecular vibrations leading thus to the possibility of highly specific fingerprinting of the molecular structure and biochemical composition of cells and tissues. Because of the inherent small Raman cross-section, superimposed with the strong autofluorescence background upon visible laser excitation of many biological media including tissue, Raman spectroscopy has limited access to trace bioanalysis. Nevertheless, in the past two decades there has been a renewed interest as a result of the nanotechnology assisted developments in surface enhanced Raman spectroscopy (SERS), which provides huge Raman signal enhancement for molecules adsorbed on specially nanostructured noble metal surfaces. Considering the sensitive detection and the ability of SERS to acquire spectral information from the close vicinity of nanoparticles, gold and silver nanoprobes can be ideal tools for the investigation of small morphological structures in cells, tissues or even living organisms [5, 6].

A critical review recently provided the latest methods for the metabolic fingerprinting using vibrational techniques as potential tools for rapid disease diagnosis, detection of dysfunction, the early intervention of therapeutic strategies, highlighting their overall conclusions that vibrational techniques could be successfully applied in such medical areas [7]. Owing to its unique ability to provide ultrasensitive detection limits, SERS has been used to detect molecular modifications involved in disease, such as cancer [8, 9], diabetes [10] and others.

Nanobiosensors are relatively new biosensing devices providing sensitive local chemical information about native biological molecules in living systems [11]. The development of nanosize labels for cell biology and biochemistry applications is currently a major research focus [12, 13]. As recently summarized, there are two main SERS configurations that have been used in biosensing [14]. An intrinsic procedure supposes that the analyte can be directly applied to the nanosurfaces and the inherent Raman spectrum of the biomolecule directly measured to identify the specimen. The extrinsic route applied the specimen immobilization onto the noble metal nanoparticles using a Raman reporter molecule to generate a signal for detection. By coating this structure with another layer of dielectrics such as SiO_2_, TiO_2_ or a polymer, a core-shell complex is formed in which the outer shell may be decorated with capture molecules such as antibodies, peptides or small molecules. When conjugated with biomolecular targeting ligands, these shell-coated nanoparticles can be used to target malignant tumours with high specificity and affinity. Thus, specimens may be captured and detected *via* a sandwich structure. This extrinsic SERS detection method has been successfully used for *in vivo* tumour targeting or rare cancer cells imaging [15, 16]. Kneipp *et al*. [17, 18] showed that gold nanoparticulates provided the optical field for ultrasenzitive probing, while also fulfilling their requirements towards the cellular system. SERS nanobiosensors based on nanoparticles provide enhanced Raman signal as a result of their local surface plasmon resonance with the applied optical field and the additional electronic interaction between the sample and the metal [19]. The implementation of nanosensors in early disease detection and subsequent treatment monitoring may result in more successful patient outcome and reduces treatment side effects [20–22].

The aim of this study was to probe the efficiency of new SERS labels based on Ag and Au nanoparticles with adsorbed cresyl violet Raman reporter molecules in cancer tissue investigation. These optical nanosensors were inoculated into different skin tissues from mice specimens with induced melanoma to probe their efficiency in SERS tissue characterization along the malignancy.

Rodents are sensitive for producing skin pathologies such as skin carcinoma by chemical tumorigenesis or endorats with proper tumour cells [23–25]. Sensitive techniques are required as early detection tools for molecular changes that are not available in routine histology [26–28].

Unlabelled Ag and Au nanoparticles were inoculated into *ex vivo* skin tissues *via* injection or by immersing the samples in the colloidal solutions. The results showed enhanced Raman signal of the tissue components in the vicinity of the uptaken nanostructures. Different signal was obtained for healthy and diseased samples. Cresyl violet (CV) chemisorbed on the Ag nanoparticles through its chromophore group resulted in double amino-functionalized nanoparticles. CV SERS labelled nanoparticles obtained in this study were successfully applied as SERS nanotags for interrogating skin tissue from *ex vivo* mice with induced melanoma.

We provide here the proof of concept in employing optical nanosensors based on SERS in interrogating the melanoma tissue from mice models. These results create perspectives for further progress in early cancer diagnostic based on amino-functionalized nanoparticles as strong Raman scattering composites, for studying tissue components *in vitro*, *ex vivo* and *in vivo*. The signal obtained from the SERS target in tissue environment provided information from the molecular components of tissue located in the close vicinity of the SERS sensor.

## Materials and methods

### Animals

C57BL/6J and Balb/c mice of 8 weeks were purchased from Charles River (Sulzfeld, Germany). Animals were maintained on tap water and diets *ad libidum* and were separated into four groups of four animals/each group. One was kept healthy and the others suffered interventions with melanoma cells and chemical carcinogens promotors/inductors. On both two types of experimental skin carcinoma/melanoma were used one group healthy, one group with pathology respectively. The work protocol followed all NIAH-National Institute of Animal Health rules: animals were maintained during the experiment in standard conditions 12 h light-dark cycle, food and water *ad libidum*, temperature 24°C and humidity above 55%. After housing for 4 weeks the mice for melanoma were inoculated subcutaneous with 0.5 × 10^6^ ml B16 melanoma cell suspension prepared just before administration with saline solution as described in cell culture protocol. The two-stage model of skin carcinoma was induced according to the following protocol: a single application of the chemical initiator mutagen 7,12-dimethylbenz[a] anthracene (DMBA) solution (0.025%–100 μl) in the first week of experiment (induction) followed by repeated applications of a pro-inflammatory phorbol ester, 12-O-tetradecanoylphorbol 13-acetate (TPA) solution (5 nM–100 μl twice a week) for 13 weeks (promotion) [29]. The solvent for chemical carcinogens was acetone (Chimopar, Bucharest, Romania). DMBA and TPA were from Fluka (Seelze, Germany). For applied methods animals were killed by cervical dislocation and the most representative skin lesions were chosen for analyses.

### Cell culture

B16 melanoma 4A5 (ECACC and Sigma-Aldrich, origin Japan stored UK) cells were grown in DME media, supplemented with 10% foetal bovine serum, penicillin (100 U/ml) and streptomycin (100 mg/ml) in specific cells culture plates. The cells were prepared in three steps (three passages respectively).

Cells were incubated with 1 ml of media at 37°C in an incubator for cell culture with 95% air and 5% CO_2_. For the subcutaneous injection with B16 cells on mice were used 0.5 ml of cells without media, in saline solution [28, 30].

Animals were killed at 14 and 25 days after implantation of cells because at this time tumours were enough in over 90% of animals (histological proved). Sections of tumours were formalin fixed. For this experiment, only the healthy mice in the incipient phase of the disease (treated with B16 melanoma cells) and one treated with 7-12 dimethylbenzantracen (DMBA) and 12-O-tetradecanoilforbol-13-acetate (TPA) cancer inductor and promoter, respectively, were used.

### Colloidal nanoparticles

Colloidal Ag and Au nanoparticles have been prepared according to classical procedures [31, 32]. In addition, the Ag colloid has been 5 min. centrifuged at 4000 rot/min.

Briefly, Ag colloidal nanoparticles were prepared by reduction in silver nitrate (AgNO_3_) with hydroxylamine hydrochloride (NH_2_OH.HCl) [31]. The final mixture had a gray-brownish colour at pH 8. The gold colloid was also prepared following an established synthesis method [32] by adding hydrogen tetrachloroaurate (HAuCl_4_) to doubled distilled water and trisodium citrate solution drop by drop while stirring. The resulting mixture was boiled and the colloidal solution obtained was of dark red colour (pH 7).

Tissues from normal, early stage melanoma (C57BL/6J mouse with B16 cells injected) and advanced melanoma (Balb/c) have been selected. Detailed experimental protocol in inducing melanoma has been reported elsewhere [29]. The SERS purpose was to evaluate the possibility to obtain spectroscopic information from diseased tissue using both labelled and unlabelled noble metal nanoparticles, therefore we do not compare here the spectral response from normal *versus* damaged tissue for reasons of minimizing the number of killed animals relative to the aim of the present work.

The SERS labels were obtained by adding 10 μl cresyl violet perchlorate (CV) 10^−4^ M aqueous solution to 1500 μl colloid, resulting in a final concentration of about 0.6 μM, whereas the nanoparticles concentration corresponding to the absorption maxima is about 2.9 × 10^10^ particles/ml.

### Histology and immunohistochemistry

**Fig. 1:**
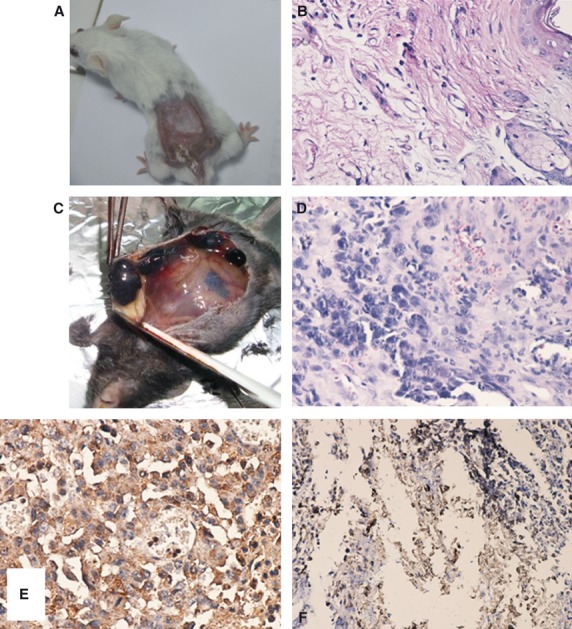
Balb/c mice and chemical carcinoma: (**A**) macroscopic image and (**B**) histological evaluation of skin (week 14) HE ×400, presence of oedematous dermis, hyperemiated blood vessels and chronically inflammatory infiltrate–strong inflammatory process and C skin subepithelial carcinoma C57BL/6J and B16 4A5 melanoma: (**C**) macroscopic image, (**D**) histological evaluation, HE ×400, diffusion of melanoma on skin level, (**E**) S100 immunohistological confirmation, Protein S-100 immunostaining malignant melanoma (×400, EnVision) and (**F**) melan-A immunohistological confirmation of skin melanoma, Positive focal reaction for melan-A in malignant melanoma (×200, EnVision).

Formalin-fixed tumour sections were H & E stained and analysed as histology samples and by application of S-100 and melan-A methods. Balb/c skin (week 14, Fig. 1A) revealed strong inflammatory process, the presence of oedematous dermis, hyperemiated blood vessels and chronically inflammatory infiltrate. The C57BL/6J mouse (B16 4A5 melanoma, Fig. 1C and D) revealed the diffusion of melanoma on skin level based on the E.S100 immunohistological confirmation.

### Statistics

Data are presented as mean and standard deviation and were analysed using one-way anova analysis. All *P* values were considered significant at the <0.05 level.

### Apparatus

The extinction spectra of the colloidal nanoparticles were recorded with a Lambda 25 double-beam scanning UV-Vis spectrometer.

The Raman and SERS spectra were acquired using two dispersive spectrometers, a DeltaNu Advantage Raman spectrometer with a USB microscope attachment, equipped with He-Ne laser operating at 4 mW, 632.8 nm, and a Bruker Senterra dispersive Raman spectrometer using the 785 nm line of a diode laser, resolution 3 cm^−1^, respectively. The laser spot size was about 3 μm.

Transmission electron microscopy (TEM) images were recorded with a JEOL JEM 1010 electron microscope (Japan Electron Optics Laboratory Co., Tokyo, Japan).

## Results

The obtained melanoma model described in the histology and immunohistochemistry section is presented in the Figure 1.

**Fig. 2:**
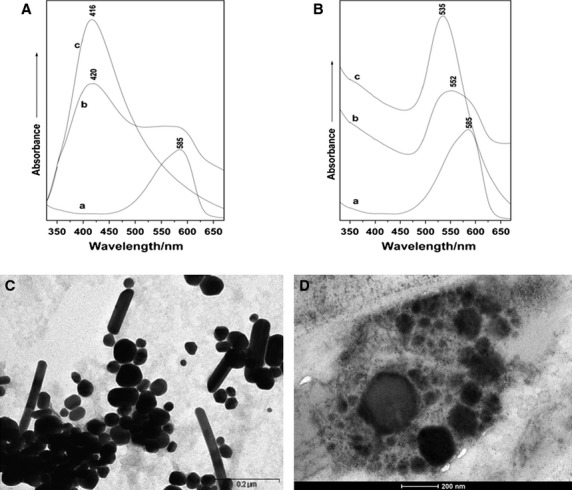
(**A**): Absorption spectra of the cresyl violet 10^−3^ M aqueous solution (a), CV-Ag nanotags (b) and the Ag colloidal nanoparticles (c). (**B**): Absorption spectra of the cresyl violet 10^−3^ M aqueous solution (a), CV-Au nanotags (b) and the Au colloidal nanoparticles (c). TEM micrographs of the Ag colloidal nanoparticles (**C**) and the section of skin sample with Ag nanoparticles uptake (**D**). Scale bar: 200 nm.

The optical properties of the metal nanoparticles were investigated using UV-Vis spectroscopy and the tunnelling electron microscopy (TEM). The Ag colloid (Fig. 2A) presents an extinction maximum at 416 nm. That provides information on the average particle size (20–50 nm) as well as additional aggregates like rods (centrifuged colloid) as suggested by TEM. Adding a small amount of the CV aqueous solution to the colloidal nanoparticles, a new broaden, red-shifted plasmonic band is observed beyond the 584 nm band of the CV absorption. This new band is caused by the nanoparticles aggregation in the presence of CV. This charge transfer band is sensitive to the analyte concentration because of the coverage effects on the Ag nanoparticles. Because of this, the intensity of the 416 nm band decreases showing that the Ag nanoparticles aggregated in the presence of the adsorbed CV molecules (Fig. 2A).

The UV-VS absorption spectrum of the Au nanoparticles (Fig. 2B) presents a maximum at 535 nm. When CV aqueous solution was added the band shape changed and shifted towards higher wavelength (from 535 to 552 nm showing further a broadening tendency). Au nanoparticles synthesized in acid conditions tend to generate particles with absorption maximum around 535 nm and the particles revealed spherical shape [33].

We evaluated the nanoparticles uptake ability of the skin tissues using transmission electron microscopy (TEM, Fig. 2C and D, respectively). Any optical evidence of nanoparticles accumulation was not observed. As suggested by the TEM images, the colloidal nanoparticles were able to penetrate the interstitial space between the cells and some of them to penetrate the cell membrane. Previous results reported by F.F. Larese *et al*. [34] found that *in vitro* nanoparticle skin permeation is possible and that silver nanoparticles as small as 25 nm stable for 24 h were located in the stratum corneum and the upper layers of the epidermis. The same passive fluid-phase uptake was accounted for the absorption of gold nanoparticles into cultured eukaryotic cells [18].

**Fig. 3:**
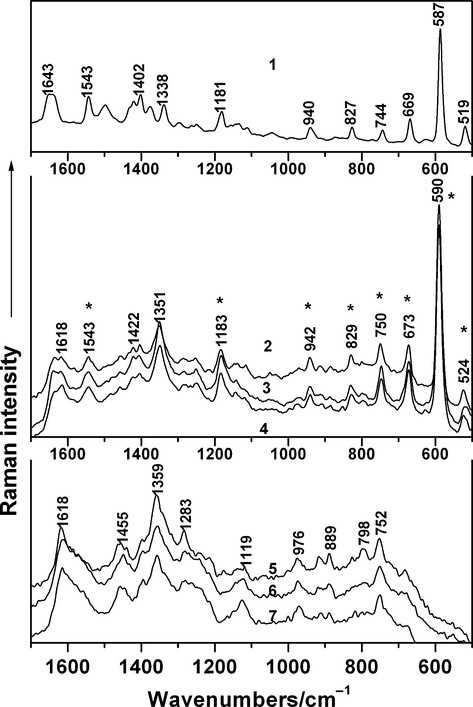
Representative SERS spectra collected from tissue (C57BL/6J mouse) using CV labelled Ag nanoparticles (2–4) as well as unlabelled nanoparticles (5–7). SERS spectrum of the label is given for eye guide (1, top spectrum). Asterisk denotes CV characteristic bands. Excitation 632.8 nm, 1 sec. integration time, four acquisitions.

Both labelled and unlabelled noble metal nanoparticles were employed to record and compare tissue SERS signal. Figure 3 presents a series of SERS spectra recorded from tissue (C57BL/6J mouse) in early stage of disease. The SERS spectral signature of the label is also presented for eye guidance. In the presence of the label we noted the intense sharp band at 590 cm^−1^ characteristic to the Raman reporter, which allowed tracking the optical nanosensors inside the tissue structure. The SERS signal from tissue with unlabelled nanoparticles is the sum of the autofluorescence background of the tissue resulted from the excitation with the visible laser line (632.8 nm) and several characteristic reproducible SERS bands located at 1618, 1455, 1359, 1283, 1119, 976, 899, 798 and 752 cm^−1^. According to Kneipp *et al*. [17] such bands are characteristic for the native chemical constituents in the cell nucleus and cytoplasm, like DNA, RNA, phenylalanine, tyrosine. Although the spectra collected from random points exhibit small band shape differences owing to the tissue inhomogeneity, the overall spectral signature is similar, thus providing a good reproducibility of the signal. The spectra are given as average signal of four acquisitions each, collected from random points of the three investigated samples.

The CV labelled nanoparticles allowed high-quality SERS spectra, as shown in Figure 3 (top). A complete vibrational characterization of the CV species adsorbed on Au or Ag revealed that its chromophore group is chemisorbed and the amine groups are less involved, resulting in double amino-functionalized nanoparticles. The CV SERS fingerprint band at 590 cm^−1^ was assigned to *in plane* skeletal quadrant stretching mode.

The spectral signature of the labelled nanoparticles inside the tissue showed the characteristic SERS bands of the label, as well as specific bands from the molecular components from the close vicinity of the label. The label bands are marked in Figure 3 with asterisk. Taking a closer look at the SERS signal around 1639 and 1351 cm^−1^ bands, we note different features compared to that from the unlabelled tissue. The amide I spectral shape band clearly shows two distinct peaks at 1618 and 1639 cm^−1^ and none of them is attributable to the label. The unlabelled tissue spectra showed one predominant band located at 1618 cm^−1^. This results suggest that the amino terminal of the label could target and bind to specific sites of some tissue components.

**Fig. 4:**
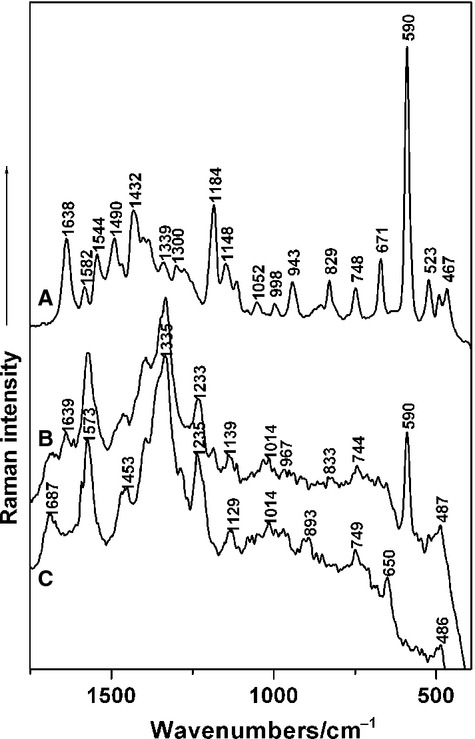
SERS spectra collected from healthy mice skin tissue (C57BL/6J mouse) with labelled (**B**) and unlabelled (**C**) Au nanoparticles. SERS spectrum of the label on the gold nanoparticles is given for eye guidance (**A**). Excitation 632.8 nm.

Surface enhanced Raman spectroscopy spectra from healthy skin using Au naoparticles are shown in the Figure 4 along with the SERS signature of the label. Pure gold nanoparticles revealed SERS (Fig. 4C) prominent bands at 1235, 1335, 1393, 1453, 1573, 1591 and 1687 cm^−1^, and weaker bands at 1129, 1014, 893, 749, 650 and 486 cm^−1^ on a strong autofluorescence background which is not completely quenched by the Au nanoparticles. CV-Au labelled tissue showed preserved major bands positions and relative intensities, however, specific differences are noted for the band shapes and relative intensities at 1233, 1286, 1591 and 1687 cm^−1^. According to Movasaghi *et al*. [35], the bands at 1687 cm^−1^ can be assigned to either amide I disordered structure or glutamic and aspartic acid. However, this band is specific for carbonyl bond that could be targeted by the amino functionals of the CV-Au label. This supposition is also supported by the observed spectral differences in the amide III region (1286 cm^−1^ band). However, further investigations are certainly needed to confirm the binding specificity of the SERS label to a particular tissue component. We also recorded for comparison a series of SERS spectra from the healthy and diseased skin in both cases of Au-CV or Ag-CV labels, respectively. Although the preparation of the colloidal nanoparticles differs, the SERS spectra acquired within just 1 sec. integration time from the same sample type are very similar (spectra not shown here). To interpret the tissue SERS signature, we compared the results with previous SERS on cells and tissue reported so far [12, 36–39]. We noted the presence of several bands that have been previously assigned preponderantly to Au-incubated cells [16, 17, 36], or to tissue [35]. Thus, the SERS label becomes a sensor for reporting certain molecular components inside the tissue. The spectral signature was basically the same for randomly chosen points on the same sample. The SERS results are supported by the TEM images that suggested detailed nanoparticles capture at the cell level (Fig. 2D) inside the tissue.

For C57BL/6J melanoma model with B164A5 cells is a clear detection of melanocyte with melanin. These appear because of the mouse strain compatibility with the parent cell line and also this cell line is a melanin containing one. The SERS investigation of the *ex vivo* skin samples showed contributions from DNA and nucleic acid bases which makes us believe that some nanoparticles are able to enter the cellular membranes. On the other hand, the disease induced by the inoculation of B16 cells into the mice specimens showed the enhancement of melanin content and the good adaptability and acceptability of skin for this B16 4A5 cell line prepared from C57BL/6J mice [28].

We supposed that the abundant melanin is responsible for signal background enhancement when visible laser line is employed. Therefore, we recorded SERS signal using a near infrared laser line to evaluate the label efficiency in surface plasmon pre-resonance condition. In NIR-SERS experiment an autopsy tissue collected from a Balb/c mice specimen treated with TPA and DMBA carcinogens was divided into three small samples, by sectioning thin transversal pieces. One sample was formaline fixed, the second was treated with formaline solution mixed with unlabelled Ag nanoparticles and one sample was immersed in formaline solution mixed with the 500 μl CV 10^−4^ M labels.

**Fig. 5:**
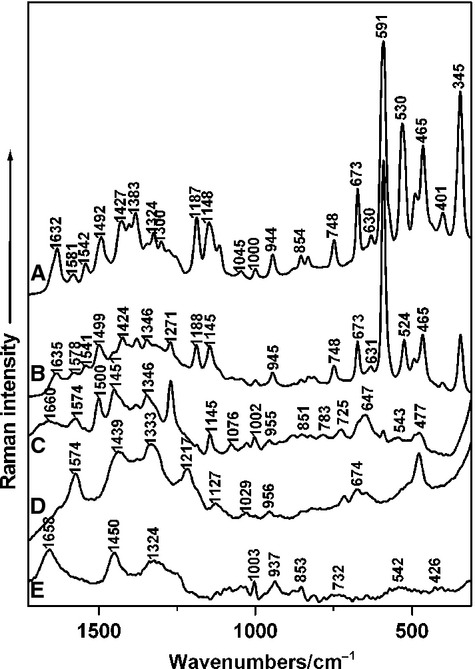
Comparative NIR-Raman and NIR-SERS spectra from the skin tissue (Balb/c): (**A**) SERS signature of the CV adsorbed on Ag nanoparticles, (**B** and **C**) SERS spectra of the labelled tissue at the surface and at 1 μm depth, respectively, (**D**) SERS spectra with unlabelled Ag nanoparticles and (**E**) Conventional Raman spectrum of the skin. Excitation 785 nm.

Figure 5 presents a comparative resume of the tissue NIR-Raman and NIR-SERS spectra. The conventional NIR-Raman spectra of the formaline-preserved skin are compared with the NIR-SERS spectra collected from tissue with CV-labelled and -unlabelled Ag nanoparticles. The observed vibrational modes are synthesized in the Table 1 together with the proposed assignment.

**Table 1:** Major Raman and SERS tissue bands observed using the NIR set-up and their proposed vibrational assignment

Raman tissue (e)	SERS tissue (d)	SERS with labelled nanoparticles 1 μm depth (c)	SERS with labelled nanoparticles at tissue surface (b)	Assignment [17, 35, 40, 43]
1658 s		1660 broad	1635 m (1632 s CV)	Amide I proteins
	1574 s	1574 m	1578 w (1582 m CV)	Guanine, adenine, ring breathing modes
			1541 w (1542 m CV)	
		1500 s	1499 s (1492 *versus* CV)	
1450 s		1451 s	1451 sh	Proteins and lipids
	1439 s			CH_2_ bending (proteins and lipids)
		1426 sh	1424 s (1427 s CV)	
		1382 w	1378 m (1383 s CV)	
		1346 s	1346 m (1346 sh CV)	
1324 m	1333 s			Guanine, DNA
		1271 s	1271 s (1277 vw CV)	Amide III band in proteins; ν C-N
	1217 s			Nucleic acids thymine
		1188 w	1188 s (1187 s CV)	
		1145 m	1145 s (1148 s)	
1123 w	1127 m			ν (C-C), ν (C-N) in lipids and proteins
		1076 m	1076 w	
	1029 w	1029 m		
1003 m		1002 m	1001 w (1000 w CV)	Phenylalanine
	956 w	955 w		ν PO_4_
			945 m (944 m CV)	
937 broad				Collagen type I
853 w				Collagen
	725 w	725 w		Adenine, ring breathing mode of DNA
	674 broad		673s (673 s CV)	Ring breathing mode of DNA bases
		591 m	591 *versus* (591 *versus* CV)	Raman label fingerprint

v: very; s: strong; m: medium; w: weak; CV: cresyl violet. (b, c, d, e): corresponds to the spectra from the Figure 5.

The NIR-Raman signal of the tissue shows vibrational modes characteristic to the main molecular components, whereas the background was substantially lower. The most intense peaks are 1658 cm^−1^ (amide III, proteins), 1450 cm^−1^ (CH_2_ bending, lipids and proteins), 1003 cm^−1^ (phenylalanine), 937 cm^−1^ and 853 cm^−1^(collagen type I). This result is in agreement with the previous reports [40] on confocal Raman investigations of murine tumour model. The NIR-SERS spectra acquired from the unlabelled tissue present most intense bands (spectrum d) usually assigned to DNA and nucleic acid bases (1574 cm^−1^, 1315–1333 cm^−1^ range). We note the amide I band absence, whereas the region 1200–1400 cm^−1^ exhibits strong SERS bands.

## Discussion

These correlations can be related to the biological observations that indicate structure changes (proteins, cholesterol, collagen and others) which are important to be quantified along the pathology. The previous reports [5] on FT-Raman skin investigation showed that all lesions, pigmentations and collagenosis influenced directly the Raman measurement. A comparison between the normal and pathologic skin (psoriatic, Kaposi sarcoma that pigmented the skin) underline that there were significant changes in the 1200–1600 cm^−1^ spectral range, including the band positions, width and relative intensity of the amide III, δCH_2_, amide II and the CH_2_, CH_3_ and OH stretching modes. The main changes were detected for collagen [41, 42].

The spectra from Figure 5 have been recorded using the NIR line at 785 nm, although the visible laser line also provided good spectra. This aspect is of particular importance in optimizing conditions for the surface plasmon resonance of the functionalized nanoparticles for tissue investigation using SERS. The red laser line falls in the wing of the CV absorption band (Fig. 2), therefore, a pre-resonance contribution to the overall enhancement would be taken into account. In the NIR excitation case, the plasmon resonance of the aggregated nanoparticles is still observed, whereas the Raman resonance of the CV is excluded. Therefore, the overall SERS signal of the marker is not dominated by the chromophore group and the functional groups supposed to bind to tissue-specific components could be better assessed from their SERS signal.

**Fig. 6:**
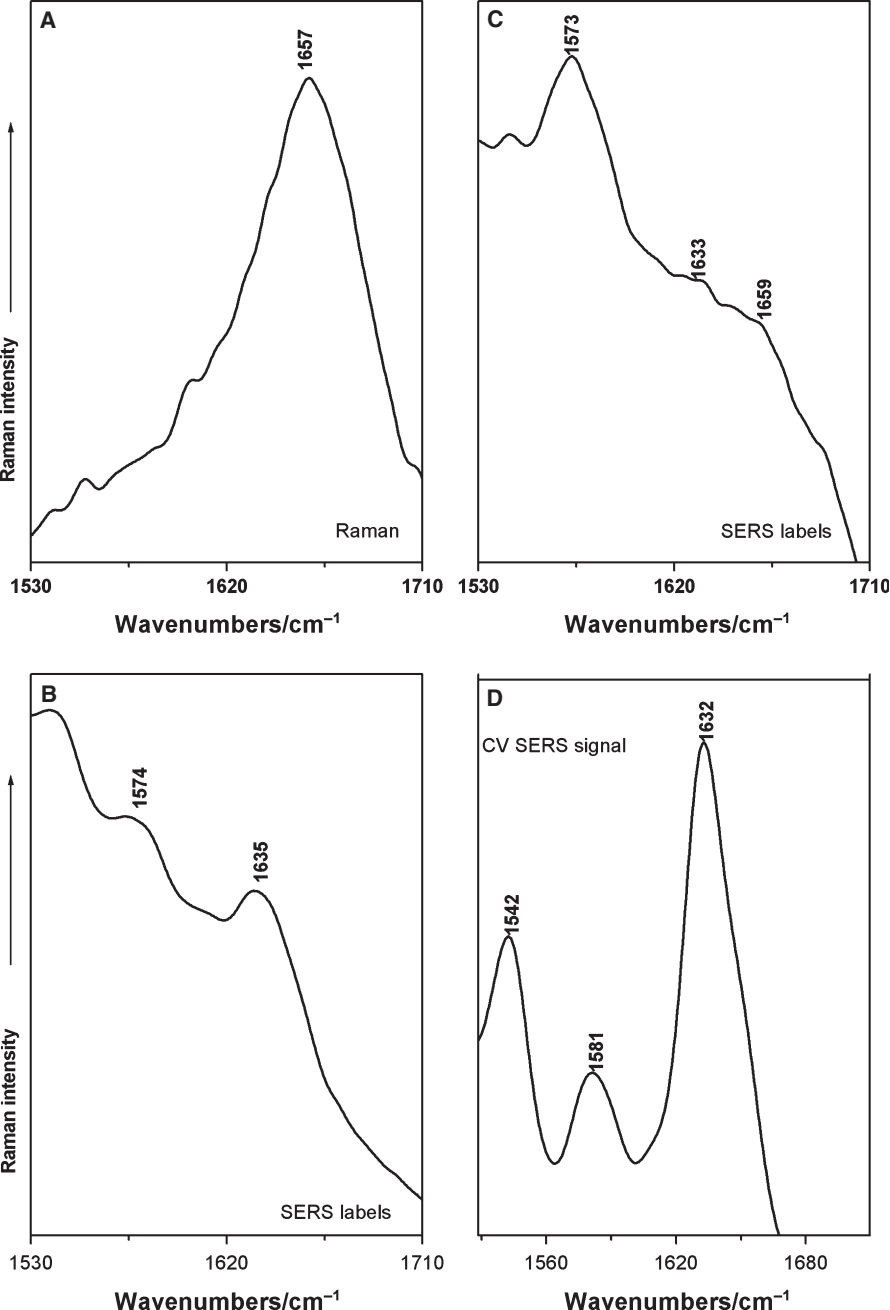
Detailed view of the amide I band from (**A**) Raman signal of the tissue samples, (**B** and **C**) SERS labels inside the tissue at the margins of the sample and in other points, respectively, and (**D**) SERS signal of the CV molecules adsorbed on the Ag nanosurface. Excitation 785 nm.

Surface enhanced Raman spectroscopy signal was sensitive to the label penetration depth (spectrum b slightly different from c). The CV label was tracked inside the tissue and recognized owing to its fingerprint bands. Besides, SERS signal from the tissue molecular components was also observed. Spectrum b) in Figure 5 resembles more with the SERS label, although weak shifts and band shape changes can be observed. Moreover, additional bands appear which are absent from both the SERS signature of the label or that of the tissue. In the high wave number range certain modifications can be noted: the appearance of new bands at 1271, 1346 and 1451 cm^−1^ and wave number shifts more than 3 cm^−1^ for the 1379, 1424, 1499 and the 1635 cm^−1^ bands. The band shape at 1635 cm^−1^ assigned to CV amino groups (3 cm^−1^ shift) changes and the relative intensity of the band decreases (Fig. 5B). The CV fingerprint at 591 cm^−1^ appears drastically decreased compared with spectrum b), whereas other CV contributions are hardly spotted because of the bands overlap with those of tissue (477, 674 or 725 cm^−1^). Other CV modes are observed at 1145, 1379 and 1574 cm^−1^. Spectrum c) collected from depth clearly showed the highly enhanced band at 1271 cm^−1^ (also observed in spectrum b, collected from the tissue surface); the 1346 cm^−1^ is enhanced and becomes broaden, probably encompassing several contributions from the skin tissue, the 1451 cm^−1^ which was previously seen as a weak shoulder is also enhanced and appears as a broad band and the 1492 cm^−1^ band of CV is shifted to 1500 cm^−1^. The amino bending of CV is distorted and a broadband centred at 1660 cm^−1^ is observed (Fig. 6C).

All these results strongly suggest an interaction between the CV amino functional groups of the labelled nanoparticles and certain tissue components. Figure 6 shows a detailed amide I spectral range, where the tissue band shape is clearly changed on passing from Raman to SERS. In addition, spectral response of the free label showed differences from that recorded in tissue, supporting our supposition. This could explain the appearance of the intense 1271 cm^−1^ band assigned to amide III and stretching vibration of C–N bonds [18].

The proof of concept in employing SERS nanosensors in interrogating the melanoma tissue from mice models is demonstrated. Upon tissue uptake of the labelled Ag and Au nanoparticles a characteristic SERS spectral feature could be obtained either in visible or NIR spectral range excitation. The nanoparticles enhanced the Raman scattering signal of the tissue molecular components found in their immediate vicinity. SERS spectra revealed dominant bands characteristic to proteins and DNA constituents indicating that some nanoparticles are located in the close vicinity of the nucleus components. SERS labels based on CV-functionalized nanoparticles proved signal specificity in different skin tissues. Such optical nanosensors can be tracked inside the tissue samples based on the spectral signature of the Raman reporter. The SERS signal reported information on either the label or the tissue molecular components from the vicinity of the label, thus such optical nanosensors hold the potential of chemical probing of tissue environment. The double amino-functionalized CV SERS labels appear to interact through their amino groups with other molecular components inside the tissue.

These results create perspectives for further progress in early cancer diagnostic based on isolating and manipulating amino-functionalized nanoparticles as strong Raman scattering composites, for monitoring specific tissue components *in vitro*, *ex vivo* and *in vivo*. The signal obtained from the SERS target in tissue environment provided information from the molecular components of tissue located in the close vicinity of the SERS sensor. Such information would be essential in understanding the molecular changes in the early cancer diagnostic, using a rapid, sensitive and non-invasive technique. Further investigations to confirm the binding specificity of the SERS label to particular tissue component as well as the molecular changes along the induced malignancy are in progress.

## References

[1] Kendall C, Isabelle M, Bayabt HF (2009). Vibrational spectroscopy: a clinical tool for cancer diagnostics. Analyst.

[2] Krafft C, Dietzek B, Popp J (2009). Raman and CARS microspectroscopy of cells and tissues. Analyst.

[3] Krafft C, Kirsch M, Beleites C (2007). Methodology for fiber-optic Raman mapping and FTIR imaging of metastases in mouse brains. Anal Bioanal Chem.

[4] Schmitt M, Popp J (2006). Raman spectroscopy at the beginning of the twenty-first century. J Raman Spectrosc.

[5] Fendel S, Schrader B (1998). Investigation of skin lesions by NIR-FT-Raman spectroscopy. Fresenius J Anal Chem.

[6] Siebert F, Hildebrandt P (2008). Vibrational Spectroscopy in Life Science.

[7] David IE, Goodacre R (2006). Metabolic fingerprinting in disease diagnosis: biomedical applications of infrared and Raman spectroscopy. Analyst.

[8] Pinzaru SC, Andronie LM, Domsa I (2008). Bridging biomolecules with nanoparticles: SERS of colon carcinoma nd normal tissue. J Raman Spectrosc.

[9] Vo-Dinh T, Allain L, David R (2002). Cancer gene detection using SERS. J Raman Spectrosc.

[10] Lyandres O, Yuen JM, Shah NC (2008). Progress toward an *in vivo* surface-enhanced raman spectroscopy glucose sensor. Diabetes Technol Ther.

[11] Vo-Dinh T, Wang H-N, Scaffidi J (2010). Plasmonic nanoprobes for SERS biosensing and bioimaging. J Biophoton.

[12] Gregas MK, Yan F, Scaffidi J (2011). Characterization of nanoprobe uptake in single cells: spatial and temporal tracking *via* SERS labeling and modulation of surface charge. Nanomedicine.

[13] Xie W, Su L, Shen A (2011). Application of surface enhanced Raman scattering in cell analysis. J Raman Spectrosc.

[14] Tripp RA, Dluhy RA, Zhao Y (2008). Novel nanostructures for SERS biosensing. Nano Today.

[15] Qian X, Peng XH, Ansari DO (2008). *In vivo* tumor targeting and spectroscopic detection with surface-enhanced Raman nanoparticle tags. Nat Biotechnol.

[16] Kneipp K, Haka AS, Kneipp H (2002). SERS in single living cells using gold nanoparticles. Appl Spectrosc.

[17] Kneipp K, Kneipp H, Kneipp J (2006). SERS in local optical fields of silver and gold nanoaggregatess. From single-molecule Raman spectroscopy to ultrasensitive probing in live cells. Acc Chem Res.

[18] Kneipp J, Kneipp H, Wittig B (2010). Novel optical nanosensors for probing and imaging live cells. Nanomedicine.

[19] Vo-Dinh T (1998). SERS using metallic nanostructures. Trends Analyt Chem.

[20] Koo H, Huh MS, Ryu JH (2011). Nanoprobes next term for biomedical imaging in living systems. Nano Today.

[21] Ray S, Reddy PJ, Choudhary S (2011). Emerging nanoproteomics approaches for disease biomarker detection: a current perspective. J Proteomics.

[22] Kasili PM, Cullum BM, Griffin GD (2002). Nanosensor for *in vivo* measurement of the carcinogen benzo[a]pyrene in a single cell. J Nanosci Nanotechnol.

[23] Alaluf S, Atkins D, Barrett B (2002). The impact of epidermal melanin on objective measurements of human skin color. Pigment Cell Res.

[24] Kollias N, Stamatas GN (2002). Optical non-invasive approaches to diagnosis of skin diseases. J Invest Dermatol.

[25] Rose ML, Madren J, Bunzendahl H (1999). Dietary glycine inhibits the growth of B16 melanoma tumors in mice. Carcinogenesis.

[26] Dehelean CA, Soica CM, Toma CC (2010). Antitumoral activity of betulin, a compound present in birch tree, in formulations with cyclodextrin. Studia Univ Vasile Goldis Life Sci.

[27] Zonios G, Dimou A, Bassukas I (2008). Melanin absorption spectroscopy: new method for noninvasive skin investigation and melanoma detection. J Biomed Opt.

[28] Roomi MW, Kalinovsky T, Roomi NW (2008). Suppression of growth and hepatic metastasis of murine B16FO melanoma cells by a novel nutrient mixture. Oncol Rep.

[29] Dwivedi C, Maydew ER, Hora JJ (2005). Chemopreventive effects of various concentrations of α-santolol on skin cancer development in CD-1 mice. Eur J Cancer Prev.

[30] Nguyen T, Novak EK, Kermani M (2002). Melanosome morphologies in murine models of Hermansky-Pudlak syndrome reflect blocks in organelle development. J Invest Dermatol.

[31] Leopold N, Lendl B (2003). A new method for fast preparation of highly SERS active silver colloids at room temperature by reduction of silver nitrate with hydroxylamine hydrochloride. J Phys Chem B.

[32] Sutherland SW, Winefordner JD (1992). Colloid filtration: a novel substrate preparation method for surface-enhanced Raman spectroscopy. J Colloid Interface Sci.

[33] Panda BR, Chattopadhyay A (2007). Synthesis of au nanoparticles at “all” pH by H_2_O_2_ reduction of HAuCl_4_. J Nanosci Nanotechnol.

[34] Larese F, Agostin FD, Crosera M (2009). Human skin penetration of silver nanoparticles through intact and damaged skin. Toxicology.

[35] Movasaghi Z, Rehman S, Rehman IU (2007). Raman spectroscopy of biological tissues. Appl Spectrosc Rev.

[36] Notingher I, Verrier S, Haque S (2003). Spectroscopic study of human lung epithelial cells (A549) in culture: living cells versus dead cells. Biopolymers.

[37] Tang HW, Yang XB, Kirkham J (2008). Chemical probing of single cancer cells with gold nanoaggregates by surface-enhanced Raman scattering. Appl Spectr.

[38] Sirimuthu NMS, Syme CD, Cooper JM (2010). Monitoring the uptake and redistribution of metal nanoparticles during cell culture using SERS spectroscopy. Anal Chem.

[39] El-Said WA, Kim TH, Kim H (2010). Detection of effect of chemotherapeutic agents to cancer cells on gold nanoflower patterned substrate using surface-enhanced Raman scattering and cyclic voltammetry. Biosens Bioelectron.

[40] Wang H, Huang N, Zhao J (2011). Depth-resolved *in vivo* micro-Raman spectroscopy of a murine skin tumor model reveals cancer-specific spectral biomarkers. J Raman Spectrosc.

[41] Dehelean CA, Soica C, Peev C (2008). Pentacyclic triterpenes interventions in skin pathology/toxicity and treatment: *in vitro* and *in vivo* correlations. Bull UASVM Vet Med.

[42] Diepgen TL, Mahler V (2002). The epidemiology of skin cancer. Br J Dermatol.

[43] Krafft C, Diderhoshan MA, Recknagel P (2011). Crisp and soft multivariate methods visualize individual cell nuclei in Raman images of liver tissue sections. Vibr Spectrosc.

